# Supplementary material for: Particulate Matter and Premature Mortality: A Bayesian Meta-Analysis
Source: Int J Environ Res Public Health. 2021 Jul 19;18(14):7655. doi: 10.3390/ijerph18147655 (PMC8303514; doi:10.3390/ijerph18147655)
Supplement: Supplementary file 1 [file ijerph-18-07655-s001.zip › ijerph-1255237-supplementary.pdf]

Online supplementary material

S1: Addressing the differences among the baseline population of the included studies:

Baseline populations in each country varied widely in relation to the numbers exposed to particulate matter. To avoid considerable disparity among studies and countries, we calculated the premature mortality rate for the respective study year by dividing the number of premature deaths by the country population of the same year. Since the premature mortality rates were not normally distributed the mortality rates were then converted to the log scale. It was then assumed that log premature mortality rates were normally distributed. Thereafter, we have tested this assumption by assessing the properties of log normal distribution were held in our log converted mortality rate data set (S2).

S2: Requirements for the Bayesian Hierarchical meta-analysis

A prerequisite for Bayesian Hierarchical meta-analysis is the normality of the data. In order to achieve normality, the log normal rates were transformed to their values in the corresponding normal distribution with the usual conversion equations which can be found in the web document [1]. In order to invoke these equations, the mean (the actual mortality rate) and variance of the log normal distribution are required. To calculate the variance, further properties of the log normal distribution were exploited [2,3]. The results of the meta-analysis were then transformed back to their original scale. (Further details in Supplementary S4&S5). Two studies were excluded from the Bayesian Hierarchical meta-analysis because they did not satisfy this criterion.

S3: The statistical analysis

The analysis was carried out in freeware R3, using the bayesmeta package version 2019 [4]. The bayesmeta package derives the posterior distributions of the synthesized mean and heterogeneity parameter and their posterior joint distribution. The Forest plots that were generated from the Bayesian hierarchical meta-analysis which demonstrated the log normal rates mapped to the normal distribution with 95% credible and prediction intervals. Thereafter the heterogeneity plots were generated to display the posterior joint density of the log normal mortality rate and heterogeneity (τ) parameters.

S4: Testing the log normal assumption

Confidence intervals for the mean of a log normally distributed variable X have the following property (equation 1), where uci and lci denote the upper and lower bound of the associated confidence intervals for the mean respectively [2,3].

$$\text{mean}^2\,(X) = \text{uci}*\text{lci} \tag{1}$$

We used a linear regression to test this assumption on the included studies' estimates. In order to fulfil the assumption of normality of errors, the square of the studies' estimated mortality rate was log transformed and the product of the mean's uci and lci was also log transformed for ease of interpretation. The coefficient obtained from the regression model was 0.992 with 95% CI (0.939, 1.045), $p < 0.0005$.

Thereafter, normality of errors and constancy of variance were assessed with graphical plots of the residuals and by formal statistical tests. The Shapiro-Wilks test for normality gave a p value of 0.47 and the Breusch-Pagan test for heteroskedasticity gave a p value of 0.47. As the value 1 is in the 95%CI for the regression coefficient, these results indicate expressed in equation 1 was met by the mortality rates and therefore we can accept that they were log normally distributed. The residual plots however, indicated the possibility of two points that may be outliers [5,6]. We further assessed these two points graphically (Figure S1). Figure S1 consists of the fitted regression line, the two lines that result from the extremities of the regression coefficient's and constant's 95% CIs and the y = x line which indicates perfect agreement with equation 1 [5,6] .

Figure S1. The results of the regression testing the assumption expressed in equation 1.

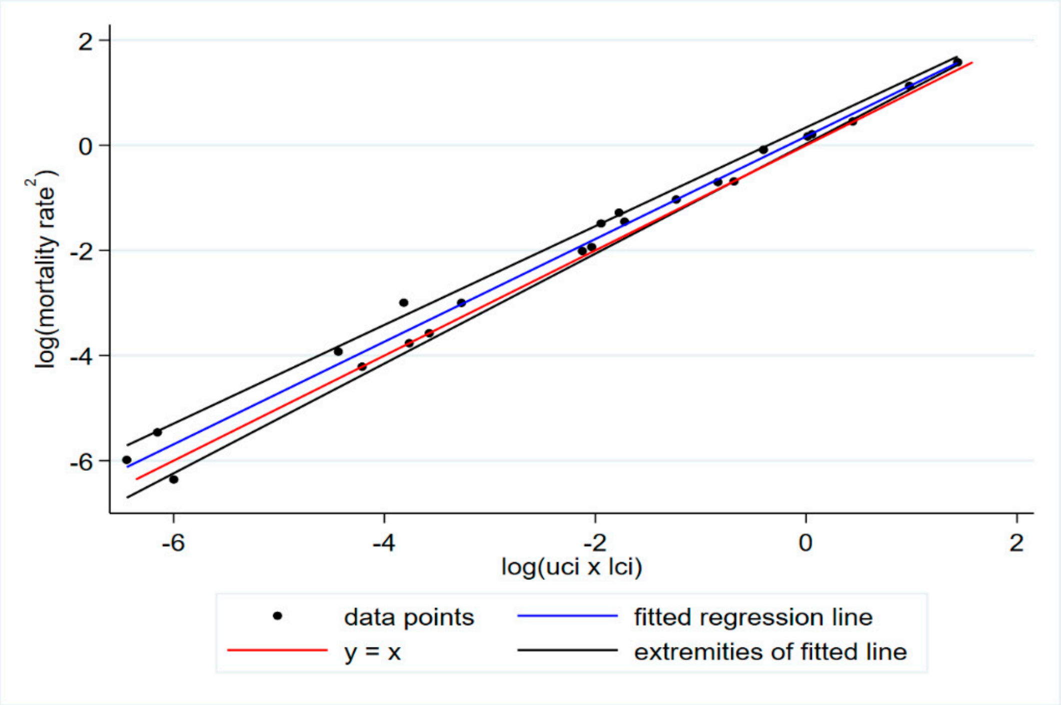

S5: Calculating the variances of the log normal distributions

The variances for the mortality rates were calculated using Equation 2 which displays a property of the variance of a log normally distributed variable X, where uci and lci are defined as in Equation 1, and z is the relative quantile point of a normal distribution required to calculate the CI. For example, for a 95% CI, z is approximately equal to 1.96 [5,6].

As the inputs to the Bayesian hierarchical meta-analysis are required to be normally distributed, the calculated log normal variance and mean (the actual mortality rate) were plugged into the conversion formulae [1] to convert the included studies' estimates to their equivalents on their associated normal distribution scales.

$$\text{Variance}(X) = \left(\frac{\text{uci}}{\text{lci}}\right)^{\frac{1}{z}} \quad (2)$$

The Bayesian hierarchical meta-analysis computes the posterior distribution of the synthesized means from which are extracted the median and a 95% credible interval based on the 2.5th and 97.5th quantiles of the posterior distribution. Please note that the synthesized mean referred to in the forest plots is the median of the posterior distribution [4]. Due to the equivariance property of the quantiles under a monotone transformation [7] and the definition of log-normality, namely that if a random variable X is log-normally distributed then the logarithm of X is normally distributed, transformation back to the original log normal scale of the median and the credible interval limits was done with exponentiation.

Reference

1. University R. Probability and statistics. 2020.
2. Aitchison JaB, J. A. C. T Cambridge University Press Cambridge The lognormal distribution : with special reference to its uses in economics 1957.
3. Limpert E, Stahel WA. The log-normal distribution. Significance. 2017;14(1):8-9.
4. RStudio Team (2020). RStudio: Integrated Development for R. RStudio, PBC, Boston, MA URL http://www.rstudio.com/.
5. Kihal-Talantikite W, Legendre P, Le Nouveau P, Deguen S. Premature Adult Death and Equity Impact of a Reduction of NO_2, PM10, and PM2.5 Levels in Paris-A Health Impact Assessment Study Conducted at the Census Block Level. International Journal Of Environmental Research And Public Health. 2018;16(1).
6. Nawahda A. Reductions of PM2.5 air concentrations and possible effects on premature mortality in Japan. Water, Air, and Soil Pollution. 2013;224(4):1508-.

7. Koenker, Roger. Quantile Regression. Cambridge University Press, 2005.
